# Genetic Regulation of DNA Methylation and Its Mediating Role in Blood Pressure: A Genome‐Wide Twin Study

**DOI:** 10.1002/mco2.71004

**Published:** 2026-09-16

**Authors:** Xuanming Hong, Ming Li, Weihua Cao, Jun Lv, Canqing Yu, Tao Huang, Dianjianyi Sun, Chunxiao Liao, Yuanjie Pang, Runhua Hu, Ruqin Gao, Min Yu, Jinyi Zhou, Xianping Wu, Yu Liu, Shengli Yin, Wenjing Gao, Liming Li

**Affiliations:** ^1^ Department of Epidemiology and Biostatistics School of Public Health Peking University Beijing China; ^2^ Key Laboratory of Epidemiology of Major Diseases Ministry of Education Peking University Beijing China; ^3^ Qingdao Center for Disease Control and Prevention Qingdao China; ^4^ Zhejiang Center for Disease Control and Prevention Hangzhou China; ^5^ Jiangsu Center for Disease Control and Prevention Nanjing China; ^6^ Sichuan Center for Disease Control and Prevention Chengdu China; ^7^ Heilongjiang Center for Disease Control and Prevention Harbin China; ^8^ Dezhou Center for Disease Control and Prevention Dezhou China

**Keywords:** blood pressure, DNA methylation, heritability, Mendelian randomization, twin study

## Abstract

DNA methylation regulates gene expression, yet its role in the genetic regulation of blood pressure (BP) remains unclear. Using 1056 Chinese National Twin Registry participants (528 twin pairs), we estimated genome‐wide DNA methylation heritability and genetic correlations between DNA methylation and BP using structural equation models. Genetically correlated CpGs were then tested using linear mixed‐effects models in the full sample and in monozygotic twin pairs. Mendelian randomization (MR) was applied using East Asian methylation quantitative trait locus (mQTL) and genome‐wide association study (GWAS) data, followed by integration of *cis*‐expression QTL and *cis*‐protein QTL data and MR‐based mediation analysis to examine whether upstream genes and proteins influence BP through DNA methylation. Genome‐wide DNA methylation heritability averaged 18.3%, with modest genetic correlations between DNA methylation and BP (mean |*r*_A_| = 0.08–0.09). Association analyses identified 914 and 624 CpGs for systolic and diastolic BP, respectively, with substantial attenuation in monozygotic within‐pair analyses, suggesting predominant genetic influences. Bidirectional relationships were identified between six CpGs and BP. MR‐based mediation estimates suggested that methylation at specific sites may mediate 34%–93% of upstream gene/protein effects on BP, implicating *HBEGF*, *CARD9*, *XCL1*, and *PCOLCE*. These findings highlight DNA methylation as a potentially important regulatory layer in genetically influenced BP pathways.

## Graphical abstract

Using 1056 Chinese twins, we estimated DNA methylation heritability (mean *h* ^2^ = 18.3%) and genetic correlations with blood pressure. Association analyses identified 914 SBP‐related and 624 DBP‐related CpGs, with attenuation within monozygotic pairs. Integrating mQTL, GWAS, eQTL, and pQTL data identified bidirectional CpG–blood pressure relationships and 13 candidate methylation‐involved pathways, supporting its role as an intermediate regulatory layer.

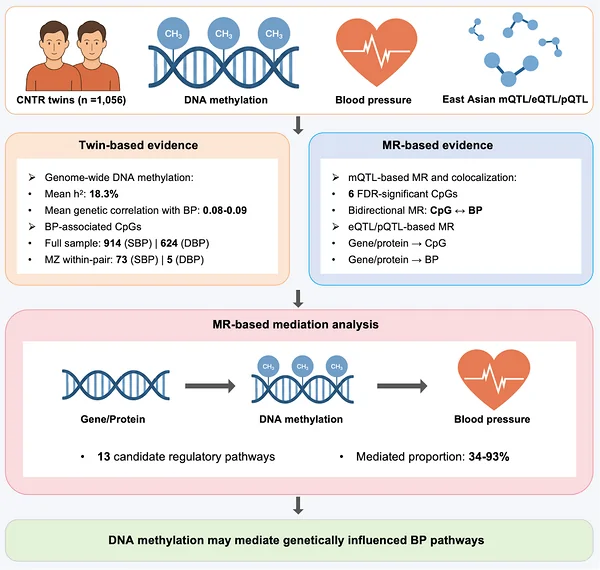

## 1 Introduction

DNA methylation is a key epigenetic modification implicated in gene regulation, primarily occurring at CpG dinucleotides [1, 2]. Gene silencing is the best known mechanism: Within promoter‐associated regulatory regions, increased methylated CpGs can suppress transcription of the associated gene [3]. Beyond transcriptional regulation, DNA methylation interacts bidirectionally with the proteome. Methylation patterns are established and maintained by proteins, including DNA methyltransferases, methyl‐CpG‐binding domain proteins, and transcription factors [4, 5]. Conversely, variation in methylation influences protein abundance by regulating the expression of protein‐coding genes. Large‐scale epigenome‐wide studies have identified hundreds of associations between DNA methylation and circulating protein levels, with methylation signatures predicting protein abundance independently of genetic effects [6].

Recently, in the context of complex trait drug development, expression quantitative trait locus (eQTL)‐ and protein QTL (pQTL)‐based Mendelian randomization (MR) has become a widely adopted framework for identifying and validating drug targets, with genetically supported targets shown to have substantially higher clinical trial success rates [7, 8]. However, the potential contribution of DNA methylation to these regulatory pathways has received comparatively little attention. This is notable given that the dynamic and reversible nature of DNA methylation makes it a potentially attractive therapeutic target [9]. A growing number of epigenetic drugs targeting the DNA methylation machinery are now in clinical development, with several already approved for clinical use [10]. Nevertheless, given their context‐dependent effects, their relevance to complex traits remains uncertain, underscoring the need to clarify the role of DNA methylation in blood pressure (BP) regulation [11].

BP is a typical major complex trait under substantial genetic regulation [12]. Hypertension, defined by persistently elevated BP, affects more than 1 billion people worldwide and remains the leading modifiable risk factor for cardiovascular disease and premature mortality [13]. Given the established genetic architecture of BP and the emerging evidence that DNA methylation interacts with genetic variation to regulate gene expression, a number of studies have examined associations between DNA methylation and BP [14, 15, 16]. Moreover, previous studies found that BP‐associated variants were enriched for associations with nearby CpG methylation [17]. Some BP‐associated CpGs were also heritable and related to gene expression [16, 18]. Integrative methylation QTL (mQTL) studies further identified candidate pathways linking genetic variation, DNA methylation, gene expression, and BP [19]. However, these studies mainly examined selected CpGs or genetically prioritized loci. Thus, the genome‐wide genetic architecture of DNA methylation and its genetic correlation with BP remain incompletely characterized, particularly in East Asian populations. Whether DNA methylation participates in pathways linking genes and proteins to BP also remains unclear.

Twin studies offer unique advantages for investigating the role of DNA methylation and underlying genetic influences in complex traits. By comparing monozygotic (MZ) twins, who share virtually all genetic variants, with dizygotic (DZ) twins, who share approximately 50%, the twin design enables formal partitioning of genetic contributions to methylation variation [20]. Crucially, this framework can extend to bivariate analyses, in which the genetic correlations between DNA methylation at individual CpG sites and a phenotype of interest can be estimated genome‐wide [21]. Such genetic correlations provide an instrument‐independent basis for identifying sites that share genetic influences with phenotypes, which cannot be directly provided by QTL‐based approaches, as they are limited to sites with available genetic instruments. These genome‐wide genetic correlations can then guide integration with downstream QTL analyses to identify potential upstream molecular factors under genetic regulation and delineate candidate regulatory pathways.

In this study, we leveraged data from the Chinese National Twin Registry (CNTR) to estimate the genome‐wide heritability of DNA methylation and to characterize its genetic correlations with BP. For CpGs with evidence of genetic correlation with BP, we conducted both full‐sample and MZ within‐pair association analyses, comparing the results to evaluate the extent to which genetic factors contribute to methylation‐BP associations. We then integrated East Asian mQTL and genome‐wide association study (GWAS) summary data with *cis*‐eQTL and *cis*‐pQTL resources to examine bidirectional relationships between DNA methylation and BP and to prioritize candidate pathways in which DNA methylation may link upstream gene expression or protein abundance to BP.

## 2 Results

### 2.1 Characteristics of CNTR

Table 1 describes the detailed demographic information of the participants. The 1056 participants had a mean age of 49.8 years (standard deviation [SD] = 12.2) and included 748 MZ twins. The mean SBP (systolic blood pressure) and DBP (diastolic blood pressure) were 136.5 mmHg (SD = 20.4) and 82.1 mmHg (SD = 11.6), respectively.

**TABLE 1:** Characteristics of the analytic samples from Chinese National Twin Registry (CNTR).

	Total	MZ	DZ
N	1056	748	308
Age, years	49.8 ± 12.2	50.4 ± 11.7	48.5 ± 13.3
Female, *n* (%)	334 (31.6)	228 (30.5)	106 (34.4)
SBP, mmHg	136.5 ± 20.4	137.2 ± 20.4	134.8 ± 20.4
DBP, mmHg	82.1 ± 11.6	82.6 ± 11.9	80.8 ± 10.7
BMI, kg/m^2^	24.8 ± 3.9	24.8 ± 3.8	24.9 ± 4.3
Antihypertensive use, *n* (%)	221 (20.9)	159 (21.3)	62 (20.1)
Smoking status, *n* (%)			
Current smoker	348 (33.0)	244 (32.6)	104 (33.8)
Former smoker	138 (13.0)	107 (14.3)	31 (10.1)
Nonsmoker	570 (54.0)	397 (53.1)	173 (56.2)
Alcohol consumption, *n* (%)			
Current drinker	448 (42.4)	310 (41.4)	138 (44.8)
Former drinker	73 (6.9)	53 (7.1)	20 (6.5)
Nondrinker	535 (50.6)	385 (51.5)	150 (48.7)

*Note*: Continuous variables are presented as mean ± standard deviation (SD).

Abbreviations: BMI, body mass index; DBP, diastolic blood pressure; DZ, dizygotic; MZ, monozygotic; SBP, systolic blood pressure.

### 2.2 Heritability of Genome‐Wide DNA Methylation

**FIGURE 1:**
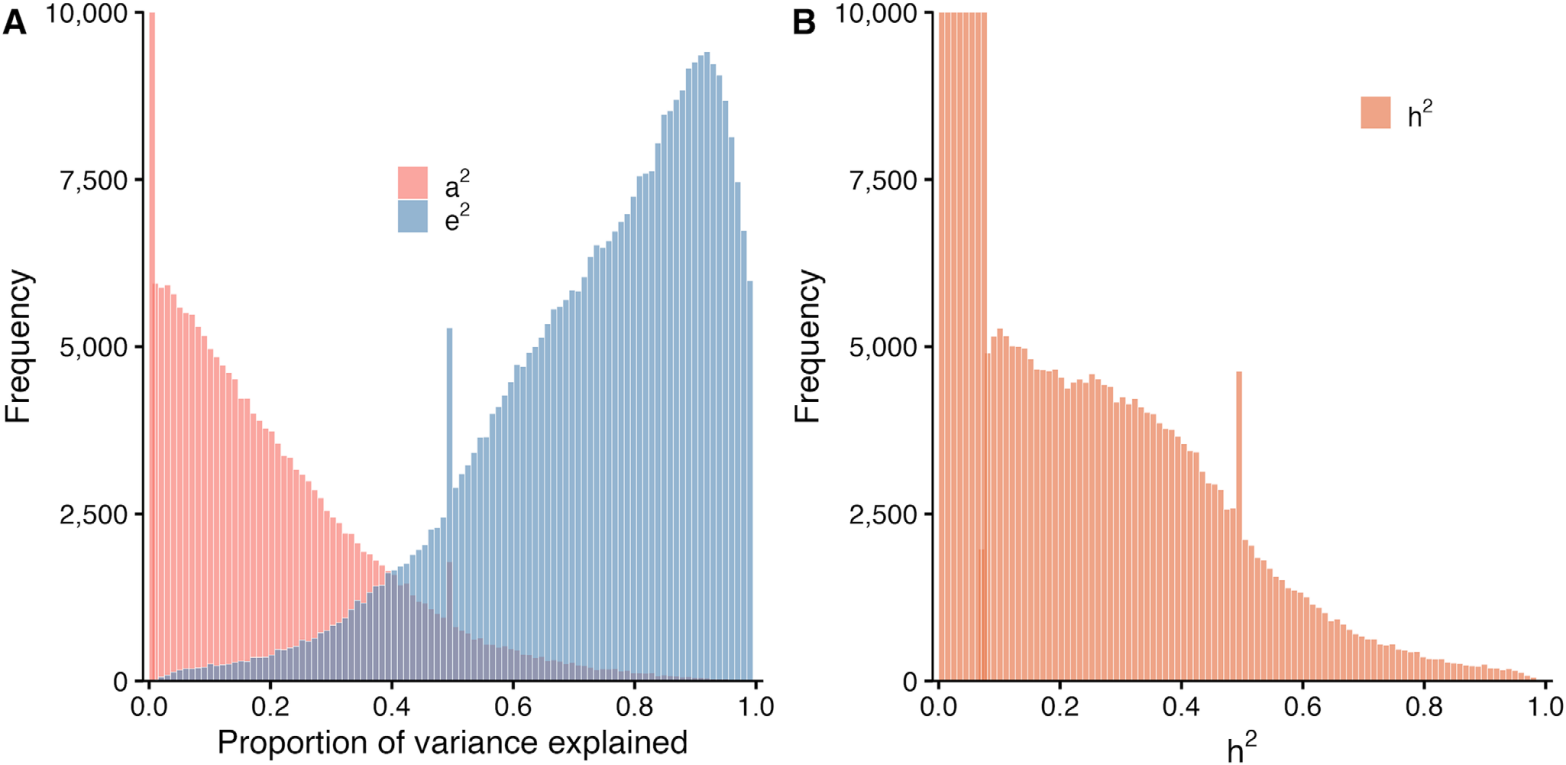
Genome‐wide heritability of DNA methylation: (A) Distribution of the proportions of variance in DNA methylation explained by additive genetic (*a* ^2^) and unique environmental (*e* ^2^) components across all CpG sites under the full structural equation model. Vertical lines indicate mean values (*n* = 948); and (B) distribution of twin‐based heritability (*h* ^2^) estimates across all CpG sites from the best fitting model (*n* = 948). *a* ^2^, additive genetic variance component; *e* ^2^, unique environmental variance component; *h* ^2^, heritability.

To characterize the genetic architecture of DNA methylation, we fitted both full (ACE or ADE) and best fitting structural equation models (SEMs) to 378,654 CpG sites. Same‐sex twin pairs were included in the SEM analyses, comprising 948 individuals. Of all tested CpGs, 209,896 sites (55.4%) were best described by the ACE model, and the remaining 168,758 sites (44.6%) by the ADE model. In the full ACE/ADE models, the additive genetic component (A) explained a mean of 9.7% (SD = 15.8%) of the variance in DNA methylation across all CpG sites (Table S1). On the basis of the best fitting submodels, the mean heritability estimate (*h* ^2^) was 18.3% (SD = 21.3%), with substantial variation across sites (Figure 1, Table S2).

### 2.3 Genetic Correlations Between DNA Methylation and BP

**FIGURE 2:**
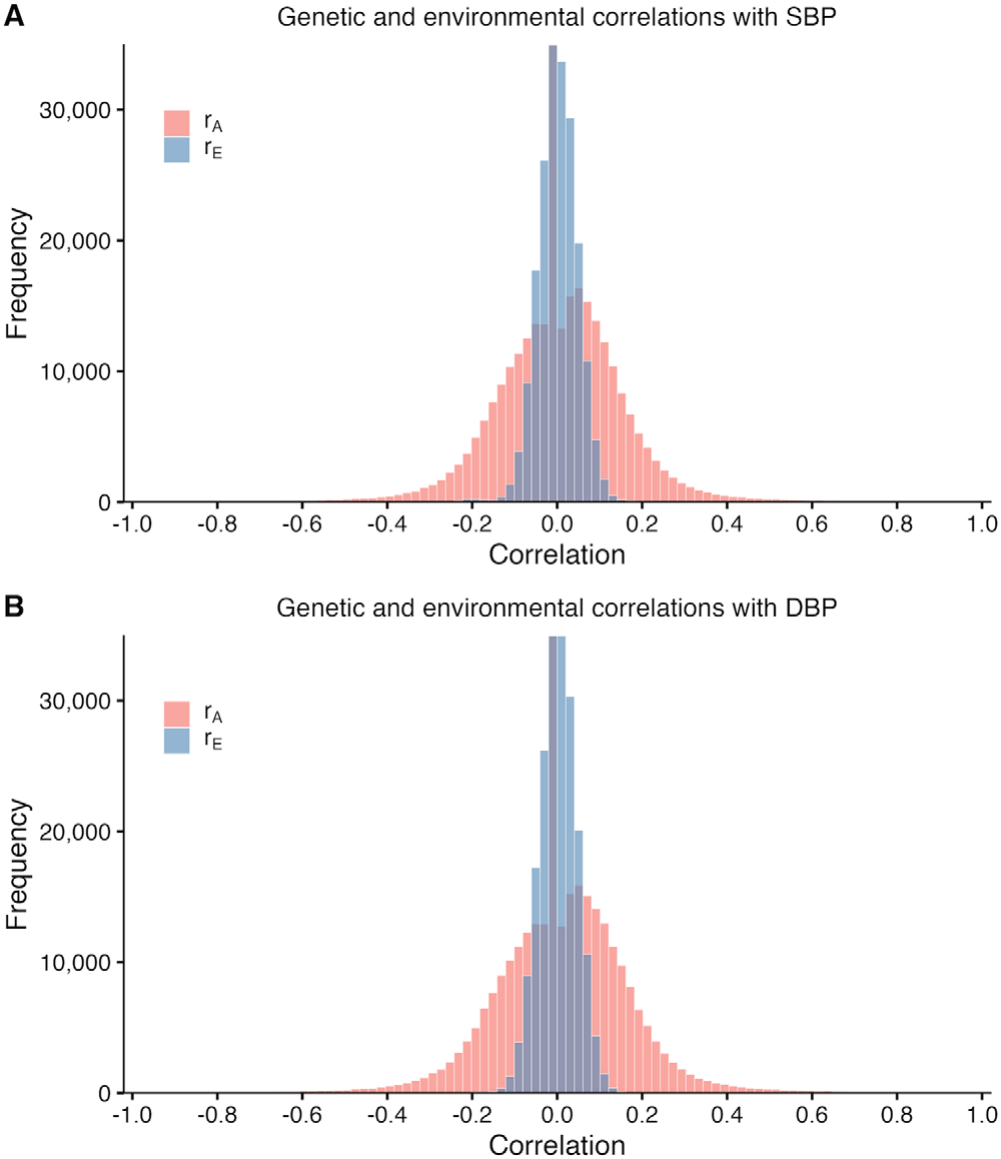
Genome‐wide genetic and environmental correlations between DNA methylation and blood pressure: Distribution of genetic (red) and environmental (blue) correlations between individual CpG sites and (A) SBP and (B) DBP (*n* = 948). |*r*_A_|, genetic correlation; |*r*_E_|, environmental correlation; DBP, diastolic blood pressure; SBP, systolic blood pressure.

We next applied bivariate SEMs to examine the genetic correlation between each CpG site and BP, providing evidence for shared genetic influences on DNA methylation and BP. The same 948 participants were included in the bivariate SEM analyses. For SBP, 151,224 CpG sites were best fitted by the ACE model and 227,419 by the ADE model. For DBP, 290,138 sites were best fitted by the ACE model and 88,514 by the ADE model. Across all genome‐wide CpG sites, the mean absolute phenotypic correlation (|*r*_ph_|) between DNA methylation and SBP was 0.04 (SD = 0.03), with a mean absolute genetic correlation (|*r*_A_|) of 0.08 (SD = 0.11). Similar patterns were observed for DBP, with a mean |*r*_ph_| of 0.04 (SD = 0.03) and a mean |*r*_A_| of 0.09 (SD = 0.11) (Figure 2, Tables S3 and S4). These results remained comparable in sensitivity analyses in which 15 and 10 mmHg were added to SBP and DBP, respectively, among participants receiving antihypertensive treatment. The mean absolute genetic correlation was 0.08 (SD = 0.10) for both SBP and DBP, and site‐specific estimates were highly consistent with those from the primary analyses (Tables S5 and S6).

Applying our predefined criteria for significant genetic correlation (|*r*_A_| > 0.2 with a 95% confidence interval excluding 0), we identified 17,643 and 23,367 CpG sites showing evidence of shared genetic influences with SBP and DBP, respectively. CpG sites meeting these criteria had a modestly higher mean *a* ^2^ of 9.8% for SBP‐correlated sites and 10.2% for DBP‐correlated sites, compared with an overall mean of 9.7%. The difference was not significant for SBP‐correlated sites (*p* = 0.15) but was significant for DBP‐correlated sites (*p* < 0.01).

### 2.4 Associations Between DNA Methylation and BP

**FIGURE 3:**
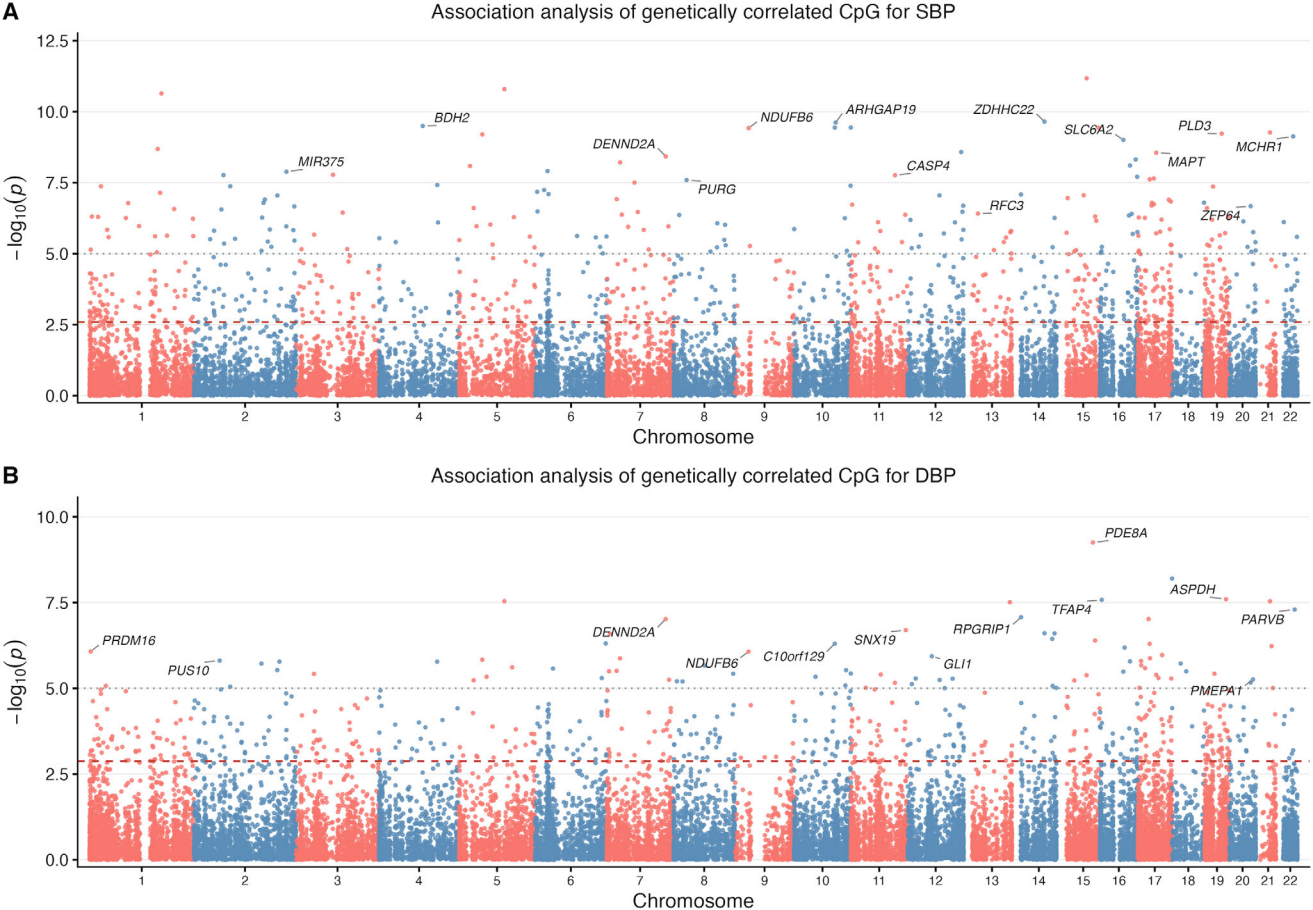
Manhattan plots of association analyses for blood pressure: Manhattan plots showing the association between DNA methylation at genetically correlated CpG sites and (A) SBP and (B) DBP (*n* = 1056). The *x*‐axis represents chromosomal position, and the *y*‐axis represents −log_10_(*p* value). The red line indicates the FDR < 0.05 threshold and the gray line indicates *p* < 1 × 10^−5^. DBP, diastolic blood pressure; FDR, false discovery rate; SBP, systolic blood pressure.

For CpG sites showing significant genetic correlation with BP (17,643 for SBP and 23,367 for DBP), we applied linear mixed‐effects (LME) models to formally test their associations with BP. In the full‐sample analysis (*n* = 1056 participants), 904 CpG sites were significantly associated with SBP and 624 with DBP (false discovery rate [FDR] <0.05) (Figure 3, Tables S7 and S8). In the within‐pair analysis of MZ twins (*n* = 748 participants), which accounts for confounding by shared genetic factors, the number of significant associations was substantially attenuated, with 73 CpG sites remaining associated with SBP and 5 with DBP (FDR < 0.05) (Figure S1, Tables S9 and S10). Of these, 63 (SBP) and all 5 (DBP) CpGs were also significant in the full‐sample analysis.

Sensitivity analyses using medication‐corrected BP values altered the number of significant SBP‐associated CpGs, decreasing from 904 to 601 in the full‐sample analysis but increasing from 73 to 153 in the MZ within‐pair analysis. In contrast, the DBP results were largely consistent with the primary analysis, with the corresponding numbers changing from 624 to 605 and from 5 to 4, respectively. Nevertheless, substantially fewer significant associations were still observed in the MZ within‐pair analysis than in the full‐sample analysis (Tables S11–S14). The marked reduction in significant associations after controlling for shared genetic background suggests that a substantial proportion of these observed methylation‐BP associations are attributable to shared genetic influences.

From the primary analyses, combining significant CpGs from both the full‐sample and within‐pair analyses and removing duplicates, a total of 914 and 624 CpGs were associated with SBP and DBP (FDR), respectively, and carried forward for subsequent MR analyses.

### 2.5 Bidirectional Associations Between DNA Methylation and BP

**FIGURE 4:**
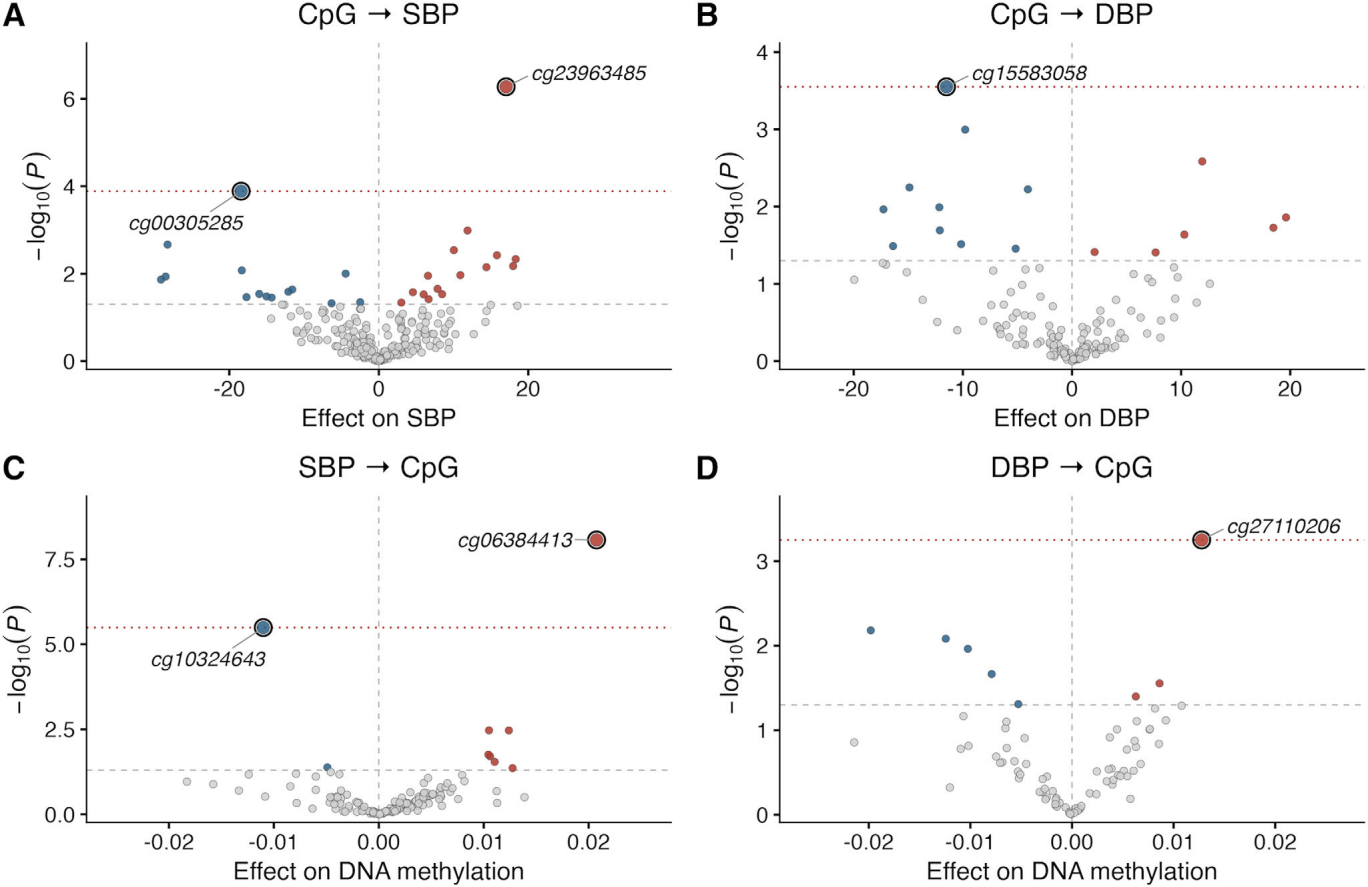
Bidirectional Mendelian randomization analyses between CpG sites and blood pressure: volcano plots showing MR estimates for (A) CpG → SBP, (B) CpG → DBP, (C) SBP → CpG, and (D) DBP → CpG. All tested associations are shown. Gray points indicate *p* ≥ 0.05, whereas red and blue points indicate nominally significant positive and negative associations, respectively. CpGs surviving FDR correction are labeled. The gray and red lines indicate *p* = 0.05 and FDR = 0.05, respectively. DBP, diastolic blood pressure; FDR, false discovery rate; SBP, systolic blood pressure.

To investigate the causal relationships between DNA methylation and BP, we performed bidirectional MR analyses for the identified BP‐associated sites. Of the 914 SBP‐associated sites, 248 had available *cis*‐mQTL instruments, of which 29 showed nominally significant effects on SBP (*p* < 0.05). After FDR correction, two sites remained significant (cg23963485 and cg00305285) (Figure 4, Table S15). Of the 624 DBP‐associated sites, 147 had available *cis*‐mQTL instruments, of which 16 showed nominally significant effects on DBP (*p* < 0.05). After FDR correction, one site remained significant (cg15583058) (Table S16). Colocalization analyses were subsequently performed for nominally significant CpG sites. Evidence of colocalization was observed for two SBP‐related CpGs, cg23963485 (PP.H4 = 0.95) and cg00305285 (PP.H4 = 0.75), and two DBP‐related CpGs, cg15583058 (PP.H4 = 0.82) and cg07160014 (PP.H4 = 0.76) (Table S17). Notably, all three CpG sites that survived FDR correction in the MR analyses were supported by colocalization.

In the reverse direction, nine CpG sites showed nominally significant evidence of being influenced by SBP (*p* < 0.05), of which two remained significant after FDR correction (cg06384413 and cg10324643) (Table S18). For DBP, eight sites showed significant associations, with one remaining significant after correction (cg27110206) (Table S19).

Given the limited number of CpG sites with available *cis*‐mQTL instruments, applying an FDR threshold at this stage would have substantially reduced the number of sites available for downstream analyses. Therefore, all significant associations (*p* < 0.05) were carried forward, and multiple testing correction was applied independently in each subsequent analytical step.

### 2.6 Effects of Genes and Proteins on BP‐Associated CpG Sites

For the CpG sites identified in the preceding MR analyses as having significant effects on BP (29 SBP‐associated and 16 DBP‐associated sites), we mapped their genomic positions and identified all genes located within ±500 kb, yielding 676 gene–CpG pairs for SBP and 326 pairs for DBP.

**FIGURE 5:**
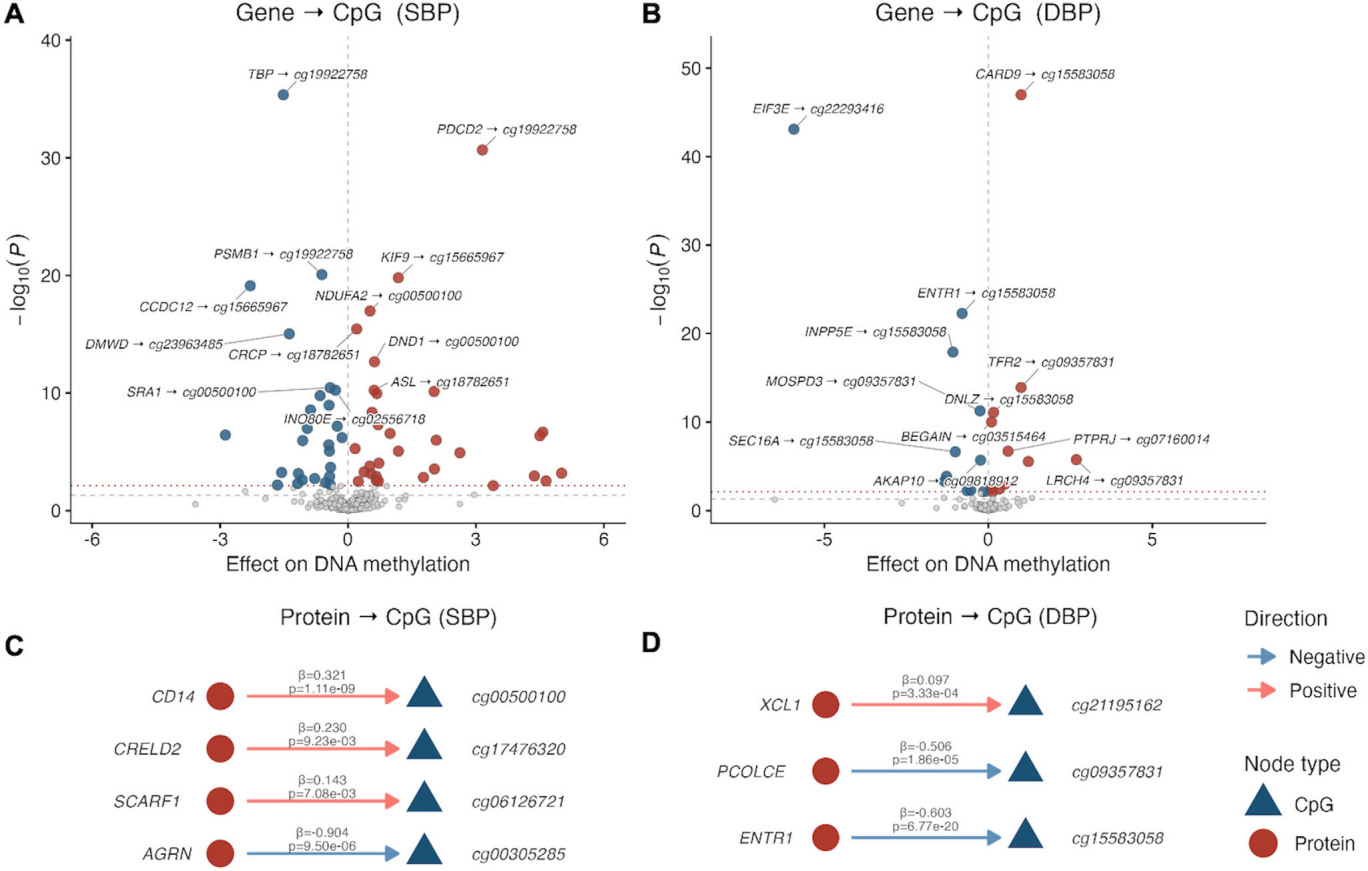
Upstream genetic regulators of blood pressure‐associated CpG sites: volcano plots showing MR estimates for gene → CpG associations in (A) SBP‐related and (B) DBP‐related pathways. Red and blue points indicate nominally significant positive and negative associations, respectively. The gray and red lines indicate *p* = 0.05 and the threshold corresponding to FDR < 0.05, respectively. All tested gene–CpG associations are shown, with the 12 associations having the smallest *p* values in each panel labeled for readability. Network plots showing protein → CpG associations for (C) SBP‐related and (D) DBP‐related pathways. Circles and triangles represent proteins and CpGs, respectively, whereas red and blue arrows indicate positive and negative effects. DBP, diastolic blood pressure; SBP, systolic blood pressure.

We first used *cis*‐eQTL data to investigate the effects of these genes on DNA methylation (gene → CpG). Of the 676 SBP‐associated pairs, 375 genes had available *cis*‐eQTL instruments. After FDR correction, 57 gene–CpG pairs showed significant effects (Figure 5, Table S20). Of the 326 DBP‐associated pairs, 132 genes had available *cis*‐eQTL instruments, and after FDR correction, 22 pairs remained significant (Table S21).

We next used *cis*‐pQTL data to investigate the effects of the encoded proteins on DNA methylation (protein → CpG). Of the 676 SBP‐associated pairs, 21 proteins had available *cis*‐pQTL instruments. After correction, four protein–CpG pairs showed significant effects: *AGRN *→ cg00305285, *CD14 *→ cg00500100, *SCARF1 *→ cg06126721, and *CRELD2 *→ cg17476320. Of the 326 DBP‐associated pairs, 16 proteins had available *cis*‐pQTL instruments (Table S22). After correction, three protein–CpG pairs showed significant effects: *PCOLCE *→ cg09357831, *ENTR1 *→ cg15583058, and *XCL1 *→ cg21195162 (Table S23).

### 2.7 Effects of Genes and Proteins on BP and Mediation Effects of Methylation

Building on the gene/protein–CpG associations identified above, we next used *cis*‐eQTL and *cis*‐pQTL instruments to examine whether these genes and proteins also exerted effects on BP.

For gene expression effects, of the 57 genes showing significant effects on SBP‐associated CpG sites, six were associated with SBP after FDR correction: *HBEGF*, *CCDC12*, *PTPN23*, *PDCD2*, *TBP*, and *FBXO46* (Table S24). Of the 22 genes associated with DBP‐related CpG sites, three were associated with DBP after FDR correction: *TFR2*, *CARD9*, and *EIF3E* (Table S25).

For protein‐level effects, of the four proteins showing significant effects on SBP‐associated CpG sites, only *AGRN* was associated with SBP after FDR correction (Table S26). Of the three proteins associated with DBP‐related CpG sites, all three showed associations with DBP and remained significant after FDR correction: *ENTR1*, *PCOLCE*, and *XCL1* (Table S27).

**FIGURE 6:**
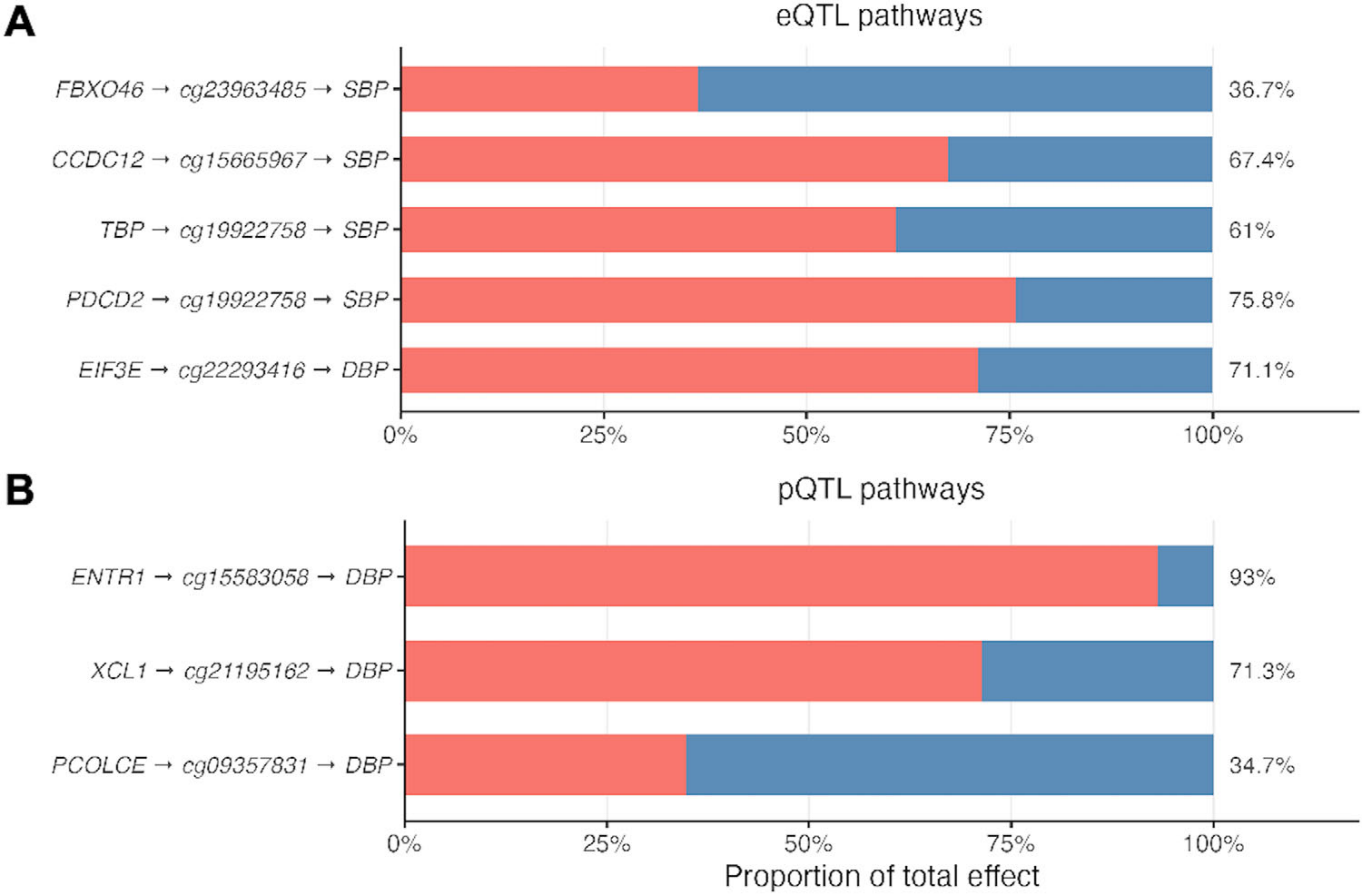
Proportion of gene and protein effects on blood pressure mediated by DNA methylation. (A) eQTL‐based gene pathways. (B) pQT‐based protein pathways. Stacked bar charts showing the proportion of the total effect of upstream genes and proteins on blood pressure mediated through DNA methylation versus the remaining direct effect. Percentages on the right indicate the mediated proportion for each pathway. Only pathways in which the gene/protein → CpG and CpG → blood pressure effects were in a consistent direction are shown. DBP, diastolic blood pressure; eQTL, expression quantitative trait locus; pQTL, protein quantitative trait locus; SBP, systolic blood pressure.

Finally, MR‐based mediation analyses were performed to quantify the extent to which DNA methylation may mediate the effects of genes and proteins on blood pressure across all identified candidate regulatory chains: gene → CpG → SBP (*n* = 6), gene → CpG → DBP (*n* = 3), protein → CpG → SBP (*n* = 1), and protein → CpG → DBP (*n* = 3). All indirect effects were statistically significant, indicating that DNA methylation may act as a mediator in these gene/protein‐BP pathways (Figure 6, Tables S28–S31).

On average, DNA methylation was estimated to mediate 67.4% of the total gene effect on SBP and 81.9% on DBP. For protein‐related pathways, the estimated proportion ranged from 34.7% to 93.0%, suggesting that DNA methylation may represent an intermediate regulatory layer through which upstream molecular signals influence BP in these specific pathways. In most pathways, the indirect effect operated in the same direction as the total effect.

## 3 Discussion

In this study, we conducted a comprehensive genome‐wide investigation of the role of DNA methylation in the genetic regulation of BP. We first estimated the heritability of DNA methylation across 378,654 CpG sites, yielding a mean *h* ^2^ of 18.3%. We then examined genome‐wide genetic correlations between DNA methylation and BP, finding mean absolute genetic correlations of 0.08–0.09, which provided initial evidence that shared genetic factors contribute to both traits. Among CpG sites with significant genetic correlations, we identified 914 and 624 sites associated with SBP and DBP, respectively. Notably, we observed a marked attenuation of significant associations in within‐pair analyses of MZ twins. Finally, integrating multi‐omics QTL data with MR, we identified bidirectional relationships between six specific CpG sites and BP. MR‐based mediation estimates suggested that DNA methylation at several loci may mediate a considerable proportion (34%–93%) of the effects of upstream genes and proteins on BP.

To our knowledge, this is the first large‐scale genome‐wide estimation of DNA methylation heritability in an East Asian population [22]. Our mean *h* ^2^ of 18.3% is consistent with the 19% reported in a large European twin cohort, suggesting that genetic influences on the methylome are broadly comparable across ancestries [20]. Similarly, the mean absolute genetic correlations of 0.08–0.09 between DNA methylation and BP are consistent with those reported between genome‐wide DNA methylation and obesity‐related traits in a parallel study [21]. Despite the modest genome‐wide averages, the substantially higher genetic than environmental correlations indicate that genetic factors are the primary driver of the observed methylation‐BP covariation. DBP‐correlated CpGs showed a modest increase in mean additive genetic contribution, whereas SBP‐correlated CpGs did not. This difference may reflect the partially distinct genetic architectures of SBP and DBP despite their strong overall genetic correlation [23]. However, this finding remains exploratory.

Notably, a key contribution of this study is the use of twin‐based genetic correlation as an instrument‐independent bridge between DNA methylation and BP. Evidence of genetic correlation can further guide downstream locus‐ and gene‐level analyses to identify potential upstream molecular factors under genetic regulation, such as gene expression, and delineate candidate regulatory pathways [24]. However, current summary‐statistic methods for estimating genetic correlation require genome‐wide association results for both traits, and their precision depends on adequate statistical power [25, 26]. Molecular QTL resources, particularly in East Asian populations, remain limited by sample size and incomplete variant‐molecular trait associations [27, 28]. Unlike QTL‐based approaches, the twin‐based framework does not require instruments for each CpG, enabling genome‐wide prioritization for downstream QTL integration when sufficiently powered summary statistics are unavailable.

On the basis of CpG sites with significant genetic correlation with BP, we first conducted association analyses and identified hundreds of methylation sites associated with SBP and DBP. Among them, two DBP‐associated CpGs, cg18933331 and cg07160014, had been previously reported [18]. Several of the top‐associated loci are annotated to genes with established roles in BP regulation. *SLC6A2* (annotated from cg03860054) encodes the norepinephrine transporter, which clears norepinephrine from sympathetic synapses. Reduced transporter activity may prolong adrenergic signaling, enhance vasoconstriction, and thereby contribute to higher SBP [29, 30]. *CASP4* (annotated from cg13128596), an inflammatory caspase that mediates vascular endothelial pyroptosis through the noncanonical inflammasome pathway, has been shown to promote pulmonary arterial hypertension and accelerate vascular inflammation [31, 32]. *PRDM16* (annotated from cg04882365 and cg22608265), a transcription factor highly expressed in vascular smooth muscle cells, was recently identified as a regulator of circadian BP variation through its control of adrenergic signaling and clock gene expression, with GWAS variants in this gene associated with SBP, DBP, and hypertension [33]. The identification of methylation sites near these genes suggests that epigenetic regulation may contribute to their expression and downstream effects on BP.

Importantly, the substantial attenuation of significant associations in the within‐pair analyses of MZ twins suggests that the observed methylation‐BP associations are largely driven by shared genetic background rather than independent epigenetic effects. This pattern is consistent with previous twin studies, which similarly found that controlling for genetic confounding substantially reduced the number of significant associations [34]. It is also biologically plausible, given that methylation at many variable CpG sites is under genetic control and enriched for mQTLs [35]. These findings may have important implications for interpreting EWAS results. CpGs detected in full population samples may reflect both genetic and nongenetic influences, whereas those retained within MZ pairs may be less dependent on shared genetic background. Although reduced statistical power may also contribute to the attenuation, the two analyses are complementary in distinguishing overall associations from those that persist after accounting for shared genetic factors.

A further important finding of this study is that mQTL‐based analyses supported bidirectional relationships between specific CpG sites and BP. Although earlier work has suggested bidirectional links between DNA methylation and BP, including genome‐wide bidirectional MR in European‐ancestry populations and cross‐lagged evidence from longitudinal twin data, our study is the first to extend this line of evidence to an East Asian context using a large blood mQTL resource [15, 18]. Biologically, these findings further suggest that DNA methylation may both contribute to BP regulation and respond to BP‐related physiological changes, pointing to a more dynamic regulatory relationship [18]. Together, these findings naturally raise the question of which upstream molecular factors drive these methylation changes.

To address this, we integrated *cis*‐eQTL and *cis*‐pQTL analyses as a candidate‐prioritization framework for tracing potential upstream drivers of the BP‐related CpG sites identified by MR, followed by MR‐based mediation analysis. Finally, we identified 13 significant MR‐based mediation effects, with consistently high estimated mediation proportions across these candidate methylation–involved gene/protein regulatory pathways (34%–93%). Several of the implicated genes are established or biologically plausible regulators of BP. *HBEGF* supports a vascular growth factor/EGFR signaling route, as HBEGF‐mediated EGFR activation is involved in angiotensin II‐related vascular signaling, vascular smooth muscle phenotypic switching, vascular remodeling, and BP homeostasis [36, 37]. *CARD9* and *XCL1* point to an immune‐inflammatory axis. *CARD9* has been shown to promote angiotensin II‐induced inflammatory and fibrotic remodeling, whereas *XCL1* fits within the broader chemokine network increasingly implicated in hypertension‐related immune‐cell recruitment, vascular dysfunction, and target‐organ injury [38, 39, 40]. *PCOLCE* highlights an extracellular matrix remodeling pathway, given its role in procollagen processing and collagen maturation, which is particularly relevant given that altered collagen turnover contributes to arterial stiffness, thereby promoting SBP elevation and the maintenance of hypertension [41]. Our findings highlight the potential involvement of DNA methylation as an intermediate regulatory layer linking upstream genes and proteins to complex phenotypes. However, as these candidate chains were inferred from MR‐based analyses, the estimated mediation proportions depend on the underlying MR assumptions and should, therefore, be interpreted with caution. At the same time, eQTL‐ and pQTL‐based analyses are now widely used in genetically guided drug target discovery to prioritize candidate targets and to strengthen causal inference for complex diseases [42]. Future studies may, therefore, benefit from considering DNA methylation alongside transcriptomic and proteomic evidence when interpreting genetically supported targets.

Several limitations should be noted. First, although we used a relatively large twin sample and East Asian multi‐omics QTL resources, the sample size remains smaller than that of large‐scale European studies. Further validation in larger datasets is, therefore, warranted given the limited availability of comparable data, particularly in East Asian cohorts. Moreover, blood‐ and plasma‐derived molecular data may not capture tissue‐specific mechanisms and require validation in tissues directly involved in BP regulation. Second, because both the mQTL and BP GWAS included participants from the Taiwan Biobank, potential sample overlap could not be excluded. Finally, although the integration of twin, MR, and mediation analyses provided a relatively consistent chain of evidence, the candidate pathways and mediation proportions were inferred from MR‐based analyses and should, therefore, be interpreted within the framework of the underlying MR assumptions. Further longitudinal and functional studies are needed to validate these findings and clarify the underlying molecular mechanisms.

## 4 Conclusions

In conclusion, this study provides a comprehensive genome‐wide evaluation of the role of DNA methylation in the genetic regulation of BP in East Asian populations. We first estimated the genome‐wide heritability of DNA methylation (mean *h* ^2^ = 18.3%) and characterized genome‐wide genetic correlations between DNA methylation and BP (mean |*r*_A_| = 0.08–0.09), providing initial evidence that shared genetic factors contribute to both traits. On the basis of CpGs showing significant genetic correlations with BP, 914 and 624 sites were identified for SBP and DBP, respectively. By integrating multi‐omics QTL data with MR, we further identified evidence of bidirectional relationships between six specific CpG sites and BP, whereas MR‐based mediation estimates suggested that DNA methylation may mediate 34%–93% of the estimated effects of upstream genes and proteins within the identified candidate pathways, highlighting DNA methylation as an important intermediate regulatory layer in genetically driven BP pathways.

## 5 Materials and Methods

### 5.1 Twin Data Source

Data analyzed in the present study were partly derived from the CNTR. The design of the CNTR, along with its recruitment strategy, data collection procedures, and participant profile, has been reported previously [43]. Data were collected during two survey waves, conducted in 2013 and 2018, and included questionnaire‐based interviews, physical examinations, and blood sample collection. For participants with eligible data from both survey waves, data from the 2018 survey were used. Ethical approval for the study was obtained from the Biomedical Ethics Committee of Peking University, Beijing, China. Written informed consent was provided by all participants, and all procedures were carried out in compliance with the Declaration of Helsinki and institutional ethical requirements (IRB00001052‐13022, IRB00001052‐14021, and IRB00001052‐22032).

Participants were eligible for inclusion if they met all of the following criteria: (1) Both members of a twin pair completed the questionnaire survey and physical examination; (2) blood samples were successfully obtained from both co‐twins; and (3) the twin pair participated in at least one detailed assessment in either 2013 or 2018. The initial sample comprised 1084 individuals, with a mean age of 49.9 ± 12.2 years. Pregnant women and their co‐twins were excluded from the study. In addition, during subsequent DNA methylation quality‐control procedures and statistical analyses, exclusion of one member of a twin pair could also result in exclusion of the corresponding co‐twin.

### 5.2 GWAS and QTL Data Sources

To characterize BP traits, we used summary statistics for SBP and DBP from a large meta‐analysis of GWAS conducted in individuals of East Asian ancestry. This meta‐analysis combined data from the Taiwan Biobank, Biobank Japan, and the East Asian subset of the UK Biobank, including 238,120 participants for SBP and 238,130 participants for DBP [44].

We also incorporated summary‐level data from mQTLs, pQTLs, and eQTL analyses. The mQTL data were derived from a recent meta‐analysis conducted in East Asian populations. This study integrated three East Asian mQTL datasets, including the Taiwan Biobank, and comprised 7619 individuals. In total, it identified and reported mQTLs for 331,048 CpG sites [45].

The plasma pQTL summary statistics were obtained from the China Kadoorie Biobank (CKB). In the CKB, plasma levels of 2923 proteins were measured in 3974 Chinese adults using the Olink platform, and genome‐wide association analyses identified 1312 *cis*‐pQTLs corresponding to 1264 proteins [46]. Summary statistics for whole‐blood *cis*‐eQTLs were derived from the Japan COVID‐19 Task Force (JCTF). This resource generated paired genotype and whole‐blood transcriptomic data from 1405 Japanese individuals. Among these, *cis* variant‐gene relationships were fine‐mapped in 1019 samples with RNA sequencing data [47].

### 5.3 Measurement of BP, Twin Zygosity, and Covariates

BP was measured on the right arm with participants in a seated position after at least 5 min of rest. Two repeated measurements were taken at an interval of more than 3 min, and their average was used for analysis. If the difference between the first two readings exceeded 10 mmHg, a third measurement was obtained, and the mean of the two closest readings was used as the final BP value. Observed SBP and DBP values were used without correction for antihypertensive medication in the primary analyses. Both the bivariate SEM and association analyses were repeated as sensitivity analyses, with 15 and 10 mmHg added to SBP and DBP, respectively, for participants receiving antihypertensive treatment.

Zygosity was determined using 59 SNPs included on the Illumina Infinium methylation arrays. Twin pairs with greater than 90% SNP concordance were classified as MZ twins [48]. A total of 816 twins (408 pairs) were categorized as MZ in this study.

Covariates included age, sex, body mass index (BMI), smoking status, and drinking status. Smoking was classified as current, former, or never [49]. Similarly, drinking was categorized as current, former, and never drinkers [50].

### 5.4 DNA Methylation Measurement

Peripheral blood samples were obtained during the same survey visit, immediately after completion of the physical examination, which included BP assessment. Genomic DNA was then subjected to bisulfite conversion using the EZ DNA Methylation Kit (Zymo Research, Orange County, CA, USA). Genome‐wide DNA methylation levels were subsequently measured using either the Illumina Infinium HumanMethylation450 BeadChip or the Infinium MethylationEPIC BeadChip (Illumina, San Diego, CA, USA).

A detailed description of methylation data processing, including signal extraction, quality‐control procedures, quantile normalization, and adjustment for blood cell composition, is provided in the Supporting Information. In brief, DNA methylation at each CpG site was quantified as a *β* value, ranging from 0 to 1, where 0 indicates complete unmethylation and 1 indicates full methylation.

Following quality control, 378,654 CpG sites shared by both the 450K and EPIC platforms were retained. A total of 1056 participants (748 MZ twins) with available DNA methylation data were included in the subsequent analyses. Among these, 474 twin pairs were with the same gender (948 individuals).

### 5.5 Genetic Instrument Selection

Genetic instruments were selected from *cis*‐QTLs for DNA methylation, protein level, and gene expression. For DNA methylation, *cis*‐mQTLs were defined as genetic variants located within ±1 Mb of the corresponding CpG site and associated with methylation levels at genome‐wide significance (*p* < 5 × 10^−8^). Variants meeting these criteria were used as candidate instruments for methylation‐related analyses. When DNA methylation was treated as the outcome, all available mQTLs from this meta‐mQTL resource were included in downstream analyses.

For plasma proteins, *cis*‐pQTL instruments were defined as variants located within ±1 Mb of the corresponding protein‐coding gene and associated with protein levels at genome‐wide significance (*p* < 5 × 10^−8^). For gene expression, *cis*‐eQTL instruments were obtained from the JCTF whole‐blood eQTL dataset and were defined as variants within ±1 Mb of the corresponding gene that reached genome‐wide significance (*p* < 5 × 10^−8^).

Across all QTL datasets, linkage disequilibrium (LD) clumping was performed to retain approximately independent variants (*r* ^2^ < 0.001). Instrument strength was further evaluated using the *F*‐statistic, and only strong instruments (*F* > 10) were retained.

### 5.6 Statistical Analysis

**FIGURE 7:**
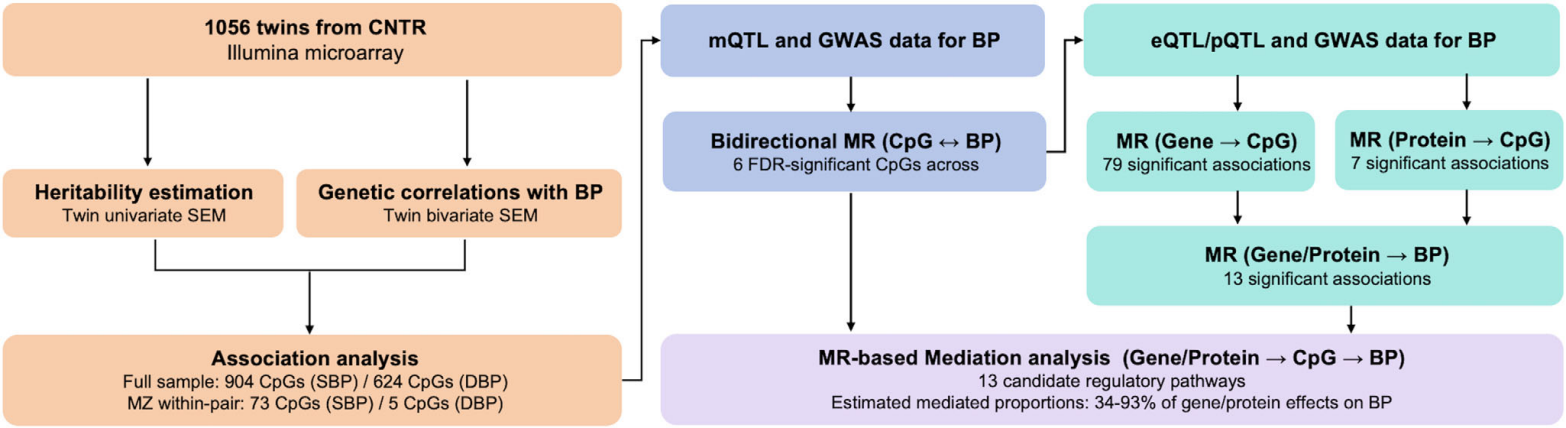
Overview of the study design and analytical framework. BP, blood pressure; CNTR, Chinese National Twin Registry; DBP, diastolic blood pressure; eQTL, expression quantitative trait locus; FDR, false discovery rate; GWAS, genome‐wide association study; MR, Mendelian randomization; mQTL, methylation quantitative trait locus; MZ, monozygotic; pQTL, protein quantitative trait locus; SBP, systolic blood pressure; SEM, structural equation model.

The analytical workflow is shown in Figure 7. All analyses were conducted using R (R statistics), version 4.3.1.

#### 5.6.1 Heritability Estimations of Genome‐Wide DNA Methylation

For each CpG site included in this study, we used SEM, implemented using the “OpenMx” package (v2.21.13), to estimate the genetic contribution to variation in DNA methylation [51]. Only same‐sex twin pairs were included in these analyses (including 948 individuals). Details of univariate SEMs in classical twin studies have been described previously [52]. Briefly, the observed variance in DNA methylation was decomposed into four latent components: additive genetic effects (A), representing the cumulative effects of alleles; nonadditive genetic effects (D), reflecting interactions between alleles; shared environmental effects (C), representing environmental influences common to both twins; and unique environmental effects (E), representing environmental factors not shared within twin pairs, together with random error. Because the C and D components cannot be estimated simultaneously in the classical twin design for twins reared together, ACE and ADE models were fitted separately [53]. The better fitting model was selected on the basis of model fit indices.

Heritability was estimated as the proportion of variance attributable to additive genetic effects (A) in the ACE model or to the combined additive and nonadditive genetic effects in the ADE model (A + D) [54]. For each CpG site, the final estimates were taken from the best fitting model identified through comparison of the full model and its nested submodels. In addition, to better reflect the cumulative contribution of allelic effects, we also report the estimate for component A from the full ACE or ADE model.

All SEM analyses were adjusted for age and sex. Model selection was based on Akaike's Information Criterion (AIC) together with one‐sided tests of variance components. Further details of the model structure are provided in the Supporting Information and Figure S2.

#### 5.6.2 Genetic Correlation Analysis Between DNA Methylation and BP

For CpG sites across the genome, we fitted bivariate SEMs to examine the extent to which the association between DNA methylation and BP could be explained by shared genetic influences [52]. In these models, the variation in DNA methylation and BP, as well as their covariance, was similarly decomposed into A, C, D, and E components, depending on the model structure. Genetic and environmental correlations, denoted as |*r*_A_| and |*r*_E_|, were derived from the variance‐covariance matrix of the bivariate SEM to quantify the extent of shared genetic and environmental influences between the two traits. As there is no established threshold for genetic correlations, significant evidence of genetic correlation between the two traits was defined pragmatically as a 95% confidence interval for |*r*_A_| that excluded 0, together with a point estimate of |*r*_A_| >0.2.

All bivariate SEM analyses were adjusted for age and sex. *p* values <0.05 were considered statistically significant. Further details of the bivariate model structure are provided in the Supporting Information and Figure S3.

#### 5.6.3 Identification of BP‐Associated CpG Sites

For CpG sites showing evidence of significant genetic correlation with BP, we next evaluated their associations with BP using LME models implemented in the “lme4” package (v1.1.36) in the full analytical sample of 1056 individuals. DNA methylation *β* values at each CpG site were treated as continuous outcome variables, and BP traits were included as predictors. BMI, age, sex, smoking status, drinking status, zygosity, and surrogate variables derived from surrogate variable analysis were included as fixed‐effect covariates. To account for the non‐independence of observations within twin pairs, twin pair ID was included as a random intercept.

To further assess the extent to which these associations might be influenced by shared genetic factors, we additionally performed within‐pair analyses in MZ twins (*n* = 748). LME models were fitted to examine the association between within‐pair differences in BP and corresponding within‐pair differences in DNA methylation. Within‐pair differences were calculated by subtracting one twin's value from that of the co‐twin. BMI, smoking status, and drinking status for each individual were included as fixed‐effect covariates, and twin pair identity was fitted as a random effect to account for within‐pair correlation [55]. To further reduce technical variation, batch effects in post‐QC DNA methylation data were corrected using the ComBat method before the discordant MZ twin analyses. Both surrogate variable analysis and ComBat correction were performed using the “sva” package (v3.54.0).

Multiple testing was controlled using the Benjamini–Hochberg FDR procedure, with statistical significance defined as FDR < 0.05. CpG annotations were obtained from the annotation file for the Illumina Infinium MethylationEPIC array.

#### 5.6.4 Two‐Sample MR

Two‐sample MR analyses were performed using summary‐level data. Before conducting MR, exposure and outcome datasets were harmonized to align effect alleles and ensure consistent strand orientation. All MR analyses were carried out using the “TwoSampleMR” package (v0.6.9). The inverse‐variance weighted (IVW) method was adopted as the main analytical approach [56]. If only one eligible instrument was available, the causal estimate was obtained using the Wald ratio [57].

Heterogeneity was assessed using Cochran's *Q* test. Multiple comparisons were accounted for by controlling the FDR. Further details of MR diagnostics and sensitivity analyses are provided in the Supporting Information.

#### 5.6.5 Bidirectional MR Between Methylation and BP

To provide mechanistic support for the relationship between DNA methylation and BP, bidirectional MR analyses were performed using summary‐level data. These analyses were restricted to CpG sites identified in the previous step as being significantly associated with BP. In the forward analysis (from methylation to BP), lead *cis*‐mQTL variants were used as instrumental variables to estimate the potential causal effects of DNA methylation on SBP and DBP. In the reverse analysis, lead independent variants associated with BP were used as instruments to evaluate the potential effects of BP on DNA methylation levels. In this setting, all available mQTLs for the corresponding CpG sites were used to represent the corresponding CpG sites.

#### 5.6.6 Colocalization Between DNA Methylation and BP

To assess whether the forward MR associations were driven by shared causal variants rather than distinct variants in LD, colocalization analyses were performed for significant CpG sites in the forward MR analyses. For each CpG site, harmonized *cis*‐mQTL and corresponding BP GWAS summary statistics within ±500 kb were analyzed using the coloc.abf function in the “coloc” package (v5.2.3). All overlapping variants within each region were included. PP.H4 represents the posterior probability that both DNA methylation and BP are associated with the region through a shared causal variant. PP.H4 > 0.75 was considered evidence of colocalization.

#### 5.6.7 *cis*‐eQTL‐ and *cis*‐pQTL‐Based MR

To investigate the potential role of DNA methylation in the molecular pathway through which gene regulation influences BP, we performed *cis*‐eQTL‐ and *cis*‐pQTL‐based MR analyses for CpG sites that showed significant effects on BP in the previous step. Genomic positions of these CpG sites were obtained from the EPIC annotation file. For each CpG site, genes located within ±500 kb, together with their corresponding encoded proteins, were identified and matched to construct CpG‐gene/protein pairs for downstream analyses. Independent *cis*‐eQTL instruments from JCTF and independent *cis*‐pQTL instruments from CKB were first used to estimate the effects of the mapped genes and proteins on DNA methylation, with all available mQTLs for each CpG site used to represent the methylation outcome. For gene/protein–CpG pairs showing significant associations, the corresponding eQTL or pQTL instruments were then evaluated against BP GWAS summary statistics to assess the effects of these genes or proteins on BP. This analytical framework was used to explore whether a putative gene/protein → CpG → BP pathway could be supported.

For gene/protein–CpG–BP pathways showing significant associations, we further conducted MR‐based mediation analyses to provide additional support for the potential regulatory role of DNA methylation. The indirect effect was estimated using the product‐of‐coefficients approach based on the MR effect estimates for gene/protein → CpG and CpG → BP, with standard errors derived using the delta method. The direct effect was calculated by subtracting the indirect effect from the total effect of gene/protein on BP, and the proportion mediated was additionally estimated for descriptive interpretation.

Detailed materials and methods can be found in the Supporting Information.

## Author Contributions

**Wenjing Gao**: conceptualization, methodology, writing – review and editing. **Ruqin Gao**: investigation. **Min Yu**: investigation. **Jinyi Zhou**: investigation. **Xianping Wu**: investigation. **Yu Liu**: investigation. **Shengli Yin**: investigation. **Ming Li**: writing – review and editing. **Weihua Cao**: writing – review and editing. **Jun Lv**: writing – review and editing. **Canqing Yu**: writing – review and editing. **Tao Huang**: writing – review and editing. **Dianjianyi Sun**: writing – review and editing. **Chunxiao Liao**: writing – review and editing. **Yuanjie Pang**: writing – review and editing. **Runhua Hu**: writing – review and editing. **Liming Li**: supervision, project administration, funding acquisition. **Xuanming Hong**: software, writing – original draft. All authors have read and approved the final manuscript.

## Funding

This work is supported by the National Natural Science Foundation of China (82373659), National Key Research and Development Program of China (2023YFC2705900), Special Fund for Health Scientific Research in Public Welfare (201502006 and 201002007), and Peking University Outstanding Discipline Construction Project of Epidemiology and Biostatistics.

## Ethics Statement

This study was approved by the Biomedical Ethics Committee at Peking University, Beijing, China (IRB00001052‐13022, IRB00001052‐14021, and IRB00001052‐22032). Informed consent was obtained from all recruited participants.

## Conflicts of Interest

The authors declare no conflicts of interest.

## Supporting information




**Supporting File 1**: mco271004‐sup‐0001‐SuppMat.docx.

**Supporting File 2**: mco271004‐sup‐0002‐Tables.xlsx.

## Data Availability

The datasets from CNTR in this study are not publicly available due to the informed consent involved in this study stating that the data of the participants could not be disclosed to a third party. But the data that support the findings of this study are available on request from the corresponding author, Prof. Wenjing Gao. The QTL and GWAS summary statistics used in this study are publicly available. The mQTL data were obtained from a recent meta‐analysis and are available at the Lin Lab website (https://lin‐lab.site/) and described in the corresponding publication (https://doi.org/10.1038/s41467‐026‐69372‐6). Whole‐blood *cis*‐eQTL summary statistics were obtained from the Japan COVID‐19 Task Force (JCTF) database (https://humandbs.dbcls.jp/en/hum0343‐v3). East Asian plasma pQTL summary statistics from the China Kadoorie Biobank (CKB) were obtained from the supplementary files of a preprint available at https://www.medrxiv.org/content/10.1101/2023.11.13.23298365v2. The GWAS summary statistics for systolic blood pressure (SBP; GCST90278668) and diastolic blood pressure (DBP; GCST90278657) are publicly available from the GWAS Catalog (https://www.ebi.ac.uk/gwas).
