# Supplementary material for: Genetic Regulation of DNA Methylation and Its Mediating Role in Blood Pressure: A Genome‐Wide Twin Study
Source: MedComm (2020). 2026 Sep 16;7(10):e71004. doi: 10.1002/mco2.71004 (PMC13583443; doi:10.1002/mco2.71004)
Supplement: Supplementary file 1 [file MCO2-7-e71004-s001.docx]

**Genetic regulation of DNA methylation and its mediating role in blood pressure: a genome-wide twin study**

**Author names and affiliations:**

Xuanming Hong^1^, Ming Li^1^, Weihua Cao^1^, Jun Lv^1^, Canqing Yu^1^, Tao Huang^1^, Dianjianyi Sun^1^, Chunxiao Liao^1^, Yuanjie Pang^1^, Runhua Hu^1^, Ruqin Gao^2^, Min Yu^3^, Jinyi Zhou^4^, Xianping Wu^5^, Yu Liu^6^, Shengli Yin^7^, Wenjing Gao^1^*, Liming Li^1^*

1 Department of Epidemiology and Biostatistics, School of Public Health, Peking University, Beijing 100191, China; Key Laboratory of Epidemiology of Major Diseases, Ministry of Education, Peking University, Beijing 100191, China.

2 Qingdao Center for Disease Control and Prevention, Qingdao 266033, China.

3 Zhejiang Center for Disease Control and Prevention, Hangzhou 310051, China.

4 Jiangsu Center for Disease Control and Prevention, Nanjing 210008, China.

5 Sichuan Center for Disease Control and Prevention, Chengdu 610041, China.

6 Heilongjiang Center for Disease Control and Prevention, Harbin 150090, China.

7 Dezhou Center for Disease Control and Prevention, Dezhou 253016, China.

**Corresponding Author:**

Wenjing Gao - Email: pkuepigwj@126.com; Postal address: Department of Epidemiology and Biostatistics, School of Public Health, Peking University, Beijing 100191, China; Phone numbers: +86 10 82801528 Ext 312

Liming Li - Email: lmlee@vip.163.com; Postal address: Department of Epidemiology and Biostatistics, School of Public Health, Peking University, Beijing 100191, China; Phone numbers: +86 10 82801528 Ext 321

**Supplementary Materials**

**Methylation data processing procedure**

Illumina Infinium Human Methylation 450K or EPIC BeadChip (Illumina, San Diego, CA, USA) were utilized to quantify genome-wide DNA methylation profiles. Specifically, the 450K array was employed on 322 samples from the 2013 survey. The EPIC BeadChip was applied for 762 samples from the 2018 survey. After blood collection, samples were centrifuged and kept at -80°C until further processing. Methylation profiling was conducted in two phases: the first batch of DNA extracted in 2016 was profiled using the Illumina 450K BeadChip, while the second batch extracted in 2020 was profiled using the Illumina EPIC BeadChip. Probes common to both array types were retained for analysis. The inter-assay reproducibility between the two BeadChip platforms has been reported to be 98%. Additionally, 90% of the probes in the 450K microarray were found to be reproducible on the EPIC BeadChip^1^. Data from the Illumina EPIC and 450K platforms were integrated using the “combineArrays” function in the minfi R package to generate a combined dataset^2^.

Methylation levels at each CpG site were expressed as β-values, representing the average methylation level spanning 0 (fully unmethylated) to 1 (fully methylated). β-values were calculated using the formula: β =M/(M+U+100), where M and U denote the average probe signal intensities at each site in the methylated and unmethylated states, respectively, assessed via the minfi R package^2^.

Quality control (QC) procedures were applied to the DNAm data to remove low-quality probes and samples. Measures included: (1) exclusion of probes showing no significant difference (P >0.01) between CpG site signals and blank controls; (2) removal of probes with minor allele frequency (MAF) > 0.01 or annotated single nucleotide polymorphisms (SNPs) on the microarray; (3) filtering of probes with detection P >0.01 in more than 1% of samples; (4) exclusion of cross-reactive probes; (5) removal of samples with probe missing rates exceeding 1%.

Subsequently, β-values were quantile normalized and adjusted for blood cell proportions (CD4T, CD8T, Mono, NK, Bcell, and Gran), estimated using the ChAMP package^3^. To minimize confounding from technical variation in DNA methylation detection, batch effects within each assay were accounted for using surrogate variable analysis in the sva R package^4^. For structural equation model and discordant MZ analyses, methylation values were corrected for known batch effects before model fitting. Batch correction was performed using the ComBat approach within the sva package.

**Univariate structural equation modeling**

The observed variance in DNA methylation was decomposed into four latent components: additive genetic effects (A), representing the cumulative effects of alleles; non-additive genetic effects (D), reflecting interactions between alleles; shared environmental effects (C), representing environmental influences common to both twins; and unique environmental effects (E), representing environmental factors not shared within twin pairs, together with random error.

As shown in Figure S2, under the ACE framework, the correlation between MZ twins reflects the combined contribution of A+C, because hypothetically, MZ co-twins share all of their genetic background, whereas the correlation between DZ twins reflects ½A+C. Variance components were evaluated by comparing the full ACE or ADE model with submodels in which specific path coefficients were constrained to zero. The better-fitting model was selected on the basis of model fit indices.

**Bivariate structural equation modeling**

In the bivariate SEMs, the variation in DNA methylation and blood pressure, as well as their covariance, was decomposed into A, C, D, and E components, depending on the model structure. To distinguish the genetic and environmental sources underlying the covariance between DNA methylation and blood pressure, a series of submodels was fitted to test whether specific cross-trait paths could be fixed to 0. For example, under the ACE model, if the cross-path representing shared additive genetic effects from DNA methylation to blood pressure (a21) could not be dropped from the model, this was taken to indicate that the two traits were influenced by overlapping genetic factors (Figure S3). The resulting genetic and environmental correlations help to clarify the contribution of shared genetic and environmental factors to the overall phenotypic correlation.

**Mendelian randomization diagnostic procedures**

**Allele harmonization**

Exposure and outcome summary statistics were harmonized to ensure effect estimates corresponded to the same effect allele and strand orientation. Palindromic variants (A/T or C/G) were handled using allele-frequency information where available. Variants with ambiguous strand orientation (i.e., allele frequencies close to 0.5) were excluded to minimize potential misalignment.

**Heterogeneity assessment**

Heterogeneity across instrument-specific causal estimates was evaluated using Cochran’s Q statistic for models with multiple instruments, to identify evidence that MR estimates varied more than expected under sampling variation alone. A nominal threshold of *P* <0.05 was used to flag heterogeneity and interpret IVW results with additional caution.

**Supplementary Tables**

Supplementary Tables S1-S31 are provided in the accompanying Excel file.

Table S1. Parameter estimates in full univariate models for genome-wide CpGs ranked by heritability (Top 300)

Table S2. Parameter estimates in best-fitting univariate models for genome-wide CpGs ranked by heritability (Top 300)

Table S3. Parameter estimates in best-fitting bivariate models for DNA methylation and SBP ranked by genetic correlations (Top 300)

Table S4. Parameter estimates in best-fitting bivariate models for DNA methylation and DBP ranked by genetic correlations (Top 300)

Table S5. Parameter estimates in best-fitting bivariate models for DNA methylation and medication-corrected SBP in the sensitivity analysis, ranked by genetic correlations (top 300)

Table S6. Parameter estimates in best-fitting bivariate models for DNA methylation and medication-corrected DBP in the sensitivity analysis, ranked by genetic correlations (top 300)

Table S7. CpG sites that passed significant threshold (FDR<0.05) to be associated with SBP in the full analysis population

Table S8. CpG sites that passed significant threshold (FDR<0.05) to be associated with DBP in the full analysis population

Table S9. CpG sites that passed significant threshold (FDR<0.05) to be associated with SBP in monozygotic twins

Table S10. CpG sites that passed significant threshold (FDR<0.05) to be associated with DBP in monozygotic twins

Table S11. CpG sites significantly associated with medication-corrected SBP (FDR <0.05) in the full-sample sensitivity analysis

Table S12. CpG sites significantly associated with medication-corrected DBP (FDR <0.05) in the full-sample sensitivity analysis

Table S13. CpG sites significantly associated with medication-corrected SBP (FDR <0.05) in the MZ twin within-pair sensitivity analysis

Table S14. CpG sites significantly associated with medication-corrected DBP (FDR <0.05) in the MZ twin within-pair sensitivity analysis

Table S15. MR results for CpG → SBP

Table S16. MR results for CpG → DBP

Table S17. CpG sites showing evidence of colocalization between DNA methylation and blood pressure

Table S18. MR results for SBP → CpG

Table S19. MR results for DBP → CpG

Table S20. MR results for Gene → CpG (SBP-associated)

Table S21. MR results for Gene → CpG (DBP-associated)

Table S22. MR results for Protein → CpG (SBP-associated)

Table S23. MR results for Protein → CpG (DBP-associated)

Table S24. MR results for Gene → SBP

Table S25. MR results for Gene → DBP

Table S26. MR results for Protein → SBP

Table S27. MR results for Protein → DBP

Table S28. Results of mediation analyses for candidate gene→CpG→SBP pairs

Table S29. Results of mediation analyses for candidate gene→CpG→DBP pairs

Table S30. Results of mediation analyses for candidate protein→CpG→SBP pairs

Table S31. Results of mediation analyses for candidate protein→CpG→DBP pairs

**Supplementary Figures**

**Figure S1. Manhattan plots of association analyses for blood pressure in monozygotic twins**

Manhattan plots showing the association between DNA methylation at genetically correlated CpG sites and (A) SBP and (B) DBP in monozygotic twins (n =748). The x-axis represents chromosomal position and the y-axis represents -log_10_(*P*). The gray and red lines indicate *P* <1×10^-05^ and the FDR <0.05 threshold, respectively.

SBP, systolic blood pressure; DBP, diastolic blood pressure; FDR, false discovery rate.

**Figure S2. Univariate structural equation model**

Squares represent observed phenotypes, and circles represent latent factors. The cross-twin correlations for additive genetic effects (A) are 1.0 for MZ twins and 0.5 for DZ twins. Those for shared environmental effects (C) are fixed at 1.0, and those for unique environmental effects (E) are fixed at 0.

**Figure S3. Bivariate structural equation model**

The correlations of additive genetic variance (A) are 1 in MZ twins and 0.5 in DZ twins. For both MZ and DZ twins, the correlations of common environmental variance (C) are 1. The cross-twin correlation for unique environmental factors (E) is 0.

**References**

1.	Pidsley R, Zotenko E, Peters TJ, et al. Critical evaluation of the Illumina MethylationEPIC BeadChip microarray for whole-genome DNA methylation profiling. *Genome Biol.* 2016;17(1):208.

2.	Aryee MJ, Jaffe AE, Corrada-Bravo H, et al. Minfi: a flexible and comprehensive Bioconductor package for the analysis of Infinium DNA methylation microarrays. *Bioinformatics.* 2014;30(10):1363-9.

3.	Tian Y, Morris TJ, Webster AP, et al. ChAMP: updated methylation analysis pipeline for Illumina BeadChips. *Bioinformatics.* 2017;33(24):3982-84.

4.	Leek JT, Johnson WE, Parker HS, Jaffe AE, Storey JD. The sva package for removing batch effects and other unwanted variation in high-throughput experiments. *Bioinformatics.* 2012;28(6):882-3.
